# The role of emotional instability in borderline personality disorder: a systematic review

**DOI:** 10.1186/s12991-023-00439-0

**Published:** 2023-03-14

**Authors:** Giulia D’Aurizio, Ramona Di Stefano, Valentina Socci, Alessandro Rossi, Tommaso Barlattani, Francesca Pacitti, Rodolfo Rossi

**Affiliations:** 1 Chair of Psychiatry, Department of Biotechnological and Applied Clinical Sciences, University of L’Aquila, Via Vetoio (Coppito 2), 67100 L’Aquila, Italy; 2 Department of Systems Medicine, University of Rome Tor Vergata, Rome, Italy

**Keywords:** Emotional instability, Emotion, Borderline personality disorder, Cognitive functions, Review

## Abstract

**Background:**

The emotional regulation process plays a pivotal role in daily-life functioning, modulating goal-directed and adaptive behavior. Conversely, altering this cognitive function can disrupt self-regulation and bring emotional dysregulation. Emotional instability could represent a core characteristic of BPD, also modulating the BPD symptom's onset. This systematic review aims to summarize the existing literature reporting the role of emotional instability in BPD to better define the role of the impairment of the emotional processes in the onset of the cognitive and behavioral symptoms of this complex mental disorder.

**Methods:**

MEDLINE, Scopus and Web of Science were independently searched for relevant studies. Eligible studies had to be identifiable through database searching, published and accessible. This systematic review was conducted according to PRISMA guidelines. The search period was from 2012 to 14 September 2022.

**Results:**

A pool of 120 studies was identified, out of which 11 met the selection criteria and were included. Overall, the studies confirm a relationship between emotional instability and borderline personality disorder.

**Conclusions:**

The evidences retrieved seem to point out the role of the emotional impairment not only in worsening of the disorder, but could also be one of the risk factors for its onset.

**Supplementary Information:**

The online version contains supplementary material available at 10.1186/s12991-023-00439-0.

## Introduction

The emotional regulation process, the analysis of the emotional stimuli, and the consequent elicited responses plays a pivotal role in human behavior [1–3]. Numerous studies have indicated how the emotional process modulates and ensures, additionally to other cognitive processes (i.e., executive functions), a goal-directed, flexible, and adaptive behavior [4–7]. Moreover, through this sophisticated cognitive function, individuals can modulate both magnitude and type of their emotional responses secondarily to the cognitive appraisal of environmental feedback [8, 9]. It follows that this process is related to normal daily-life functioning. Conversely, an alteration of the emotional process can disrupt the self-regulation process and bring out emotional dysregulation. A large body of literature has highlighted how a prolonged alteration of this cognitive process can cause emotional distress and severe mental disorders, including anxiety and mood disorders [10–12], post-traumatic stress disorder [13, 14], eating disorder, and substance abuse [15, 16].

Diagnostic and statistical manual of mental disorders, 5th edition (DSM-V) defines the construct of emotional instability or affective lability, as “unstable emotional experiences and frequent mood changes; emotions that are easily aroused, intense, and/or out proportion to events and circumstance” [17], including it in the affective diagnostic criteria for borderline personality disorder (BPD).

According to Putnam and Silk [18], emotional instability, or dysregulation, could represent a core characteristic of BPD, also modulating the predisposition for the BPD symptoms onset [19–21]. The clinical features of BPD are characterized by a specific pattern of signs including the dysregulation of the interpersonal relationship, impulse control, impulse aggression, suicidal tendency, and pervasive emotional instability [22]. Typically, in this disorder, the alteration of the emotional process results in a strong and negatively connoted affective response that includes terror, panic, shame, pain, and anger [23]. Moreover, patients experiencing a dysfunctional mood reactivity, rapidly switch from dysphoric states to euthymia and vice versa [24], as well as an over-reactivity and sensitivity to emotional trigger [25]. Additionally, similarly to other neurological and mental disorders also characterized by emotional dysfunction [26–28], in BPD emotional instability does not allow engage adaptive strategies of emotion regulation, freezing individuals to dysfunctional behaviors and maladaptive coping strategies (i.e., impulsive suicidal behaviors, rumination, thought suppression) [29].

In addition to emotion instability, interpersonal impairment, and impulsiveness, the dysregulation in metallizing process has been proposed as a core domain in BPD [30, 31]. Typically, mentalizing is referred to as the ability to understand the mental state of one's own and others and which one can explain the behavior of others and ourselves [32]. Conversely, an alteration of this cognitive process can result in impairment in mindreading ability, also dysregulated in the BPD [33]. Therefore, this process has a strong adaptive meaning, also modulating both social and interpersonal interaction.

According to Fogany and colleagues [34], impaired mentalization is central in BPD. Similarly, Bateman and Fonagy [35] argue that a dysfunction in the mentalizing capacity, which could underlie the poor quality of social interactions in patients with BPD, is a core feature of this mental disorder and also results in an impairment in the ability to elaborate emotional stimuli, emotional regulation, and managing impulsivity. It follows that promoting and stimulating the development of mentalizing in BPD through structured intervention protocols can improve the capacity of the patients to self-regulate their emotional states with positive consequences on interpersonal and social interaction and, therefore, on the quality of one’s life.

In light of this, given the key role that both emotions and the correct processing of emotional environmental stimuli have in the regulation of individual behavior and interaction with others, it is essential to clarify how these cognitive processes are dysregulated in those mental illnesses in which they represent one of the most invalidating aspects.

In the present systematic review, we aimed at summarizing the existing literature reporting the core role of emotional instability in BPD, highlighting the impact that the dysregulation of the emotional processes have on the onset of the cognitive and behavioral symptoms of BPD and how the impairment of the ability to processing emotion stimuli can negatively alternate the global functioning of the patients with BPD. Considering the pervasive role of emotional instability in the stabilization of this mental disease and the consequent impact on the patient's quality of life, bringing out the specificity of the dysregulation of this cognitive process can promote the development of ad hoc treatment for BPD.

## Methods

We conduct a systematic review according to “Preferred Reporting Items for Systematic Review and Meta-Analyses” (PRISMA) guidelines which were update in 2020 [36].

### Search strategy and study selection

We combined the keywords “emotional instability”, “emotional dysregulation”, and “borderline personality disorder” joined by the Boolean operator AND, and searched on Scopus, MEDLINE, and Web of Science. The search period was from 2012 to 14 September 2022.

In a two-step blinded process, 3 raters (D.A.G., D.S.R. and B.T.) selected the records. Firstly, the raters examined the title and abstract and, subsequently, based on the reference's eligibility, they examined full references. In the article selection process, all records were assessed by the raters, and, if they were not in agreement, the article was not included in the systematic review.

### Inclusion criteria

The full text was considered eligible if respecting the following criteria: 1) to be a primary study fully published (case reports and series, reviews, and editorials were not included); 2) to include patients with BPD; 3) to investigate the construct of emotional instability in BPD. The search was limited to English-language publications and human samples.

### Exclusion criteria

Conducting the reports analysis, we excluded studies which were defined not relevant to the aim of the systematic review. Moreover, all preprint and incomplete or unpublished studies were not included in this systematic review. Design studies such as “letter”, “feature article”, “commentary”, “short communication”, and “abstract”, were excluded as well.

### Data extraction and analysis

Data extraction was performed independently by D.A.G., D.S.R. and B.T. S.V. approved the selected articles. The researchers used a Microsoft Excel electronic spreadsheet to extract and collect the following data: (1) first author name; (2) journal; (3) year of publication; (4) study design; (5) objectives of the study, sample size; (6) questionnaire of assessment; (7) main study finding. Finally, extracted data were stored in a computerized database.

### Quality assessment

The quality of studies was evaluated independently by D.A.G. and D.S.R. and S.V. using Newcastle Ottawa Scale (NOS). Potential discrepancies during article selection were solved by confrontation. Finally, studies found to be non-satisfactory according to NOS tool were excluded.

## Results

### Literature search

**Fig. 1:**
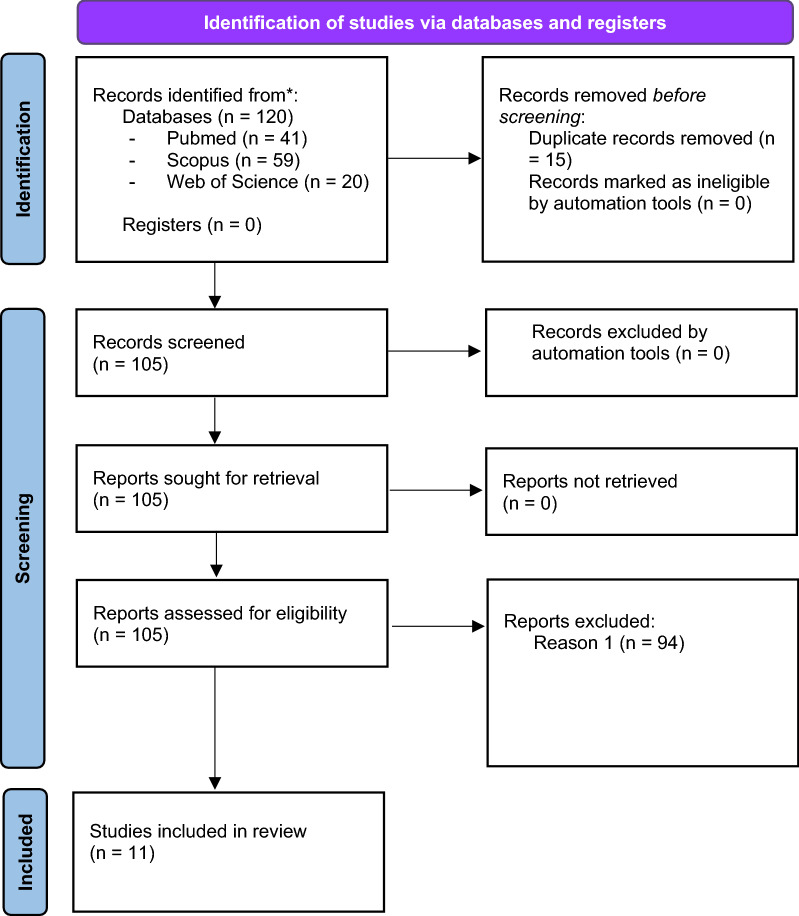
PRISMA flowchart of included studies

The database search identified 120 records. After duplicate removal, 105 articles were screened for eligibility. Secondarily to this step, 94 articles were excluded. The full text of the remaining articles was examined. Finally, 11 articles [37–47] published from 2012 to 2022 were included in the database and considered for this review. The process of the study selection is illustrated in Fig. 1.

The search line syntax and the main characteristics of the assessed studies are shown in Additional files 1, 2.

The search was focused on the role of emotional instability in BPD, especially on the intrinsic features that this cognitive dysregulation assumes in this mental disorder.

Martino et al. gathered data from the comparison between 44 younger and 49 older BPD patients, founding that, in general, both groups reported similar levels of emotional dysregulation and impairment of social functioning. Moreover, the similar dysregulation in emotional process obtained from older and younger groups showed severe impairment in the emotion regulation strategies. This seems to confirm how, independently the age of the patient, in BPD, exists a pervasive and time-independent emotional instability. According to the authors, emotional instability could be configured as a stable feature in BPD patients. Therefore, emotional dysfunction will lead to errors in the processing of emotional stimuli and to non-adaptive behaviors [37].

The over the time stability of the emotional dysregulation in BPD is also reported in a longitudinal study in which the authors have investigated this factor in BPD patients over the course of 12 months. Specifically, Stepp and colleagues have hypothesized that the development trajectory of emotional impairment, in this time window, can predict global BPD features. The results showed an increase in emotional dysregulation over time and how this factor fully mediates the BPD severity symptoms at baseline and at 12-month follow-up. These specific emotional impairment features define emotional instability as an underlying factor and a maintenance mechanism of the BPD [38].

In a longitudinal study to investigate the potential predictors of the poor clinical course of BPD, Yen et al. found that, in general, the number of BPD symptoms negatively predicts the clinical disease course (OR: 1.43; 95% CI 1.05–1.95; *p*: 0.02). Interestingly, stronger association, in this analysis model, emerges when considering the affective dysregulation factor (OR: 1.49; 95% CI 1.13–1.98; *p*: < 0.01). These results confirm the centrality of the emotional dysregulation process in BPD [39].

In daily-life functioning, individuals with BPD experiment with unstable interpersonal relationships. Altered and non-adaptive emotional responses to interpersonal and environmental stressors, associated with emotional instability, may explain this behavior and psychological impairment. This feature of BPD becomes even more evident when subject has to process negative stimuli (i.e., stressors). In this situation, individuals with BPD show a slow return to baseline. This is what Hepp et al. revealed explaining how an altered ability to regulate emotional responses and manage interpersonal stressors may contribute to an increase in the severity of BPD symptoms [40].

Houben et al. proposed that the emotional instability of BPD can be explained by an "emotional switching" process consisting of a rapidly and strong change between negative and positive emotions that occur in daily functioning. Analyzing data from 30 BPD patients and 28 controls, the authors found a specific effect that explains how affective instability in BPD patients is characterized by a larger "emotional switching" from negative to positive emotional states and vice versa when compared with controls [41].

Interestingly, in another study by Houben and colleagues, the aforementioned “emotional switching” also emerges in the other clinical groups investigated. (i.e., bulimia nervosa and post-traumatic stress disorders). [42].

Kobeleva et al. highlighted the negative role that unstable relationship, non-adaptive anger response, and affective instability has in the daily life of individuals with BPD, found a significant difference between BPD and control groups showing emotion-specific alteration during the evaluation of happy and fearful faces in the BPD patients [43].

Dick & Suvak investigated the possible relationship between language-based variables (i.e., vocabulary, semantic arousal, and valence focus) and emotional responses. The study found that language ability positively modulates the emotion generation process, highlighting how language is a pivotal factor in the emotional instability and how a vocabulary increased competence could play a protective role mitigating the negative effect of emotional process dysregulation in BPD [44].

Regarding the relationship between the severity degree of emotional dysregulation and cognitive impairment, Liu and colleagues examine how emotional instability modulates the working memory (WM) performance on BPD by using event-related potentials (ERPs). Despite no differences found on behavioral results between BPD patients and control subjects, the lower P3 amplitude and longer N2 latency in BPD group seemed to reveal an altered neural activity in WM sub-components [45].

Ruocco et al. [46] conducted a meta-analysis exploring the neural dysfunction that underlines emotional alteration in BPD. The results indicated that BPD patients, with respect to control subjects, showed greater neural activation both in the insula and posterior cingulate cortex. Conversely, patients showed less activation than the control group in a neural network including amygdala-dorsolateral PFC.

Taking into account the centrality of emotional instability in BPD, Richetin et al. [47] conducted a network analysis on the 9-criteria BPD structure to explore their possible relationship both in BPD and control groups. Results confirm that emotional dysfunction, with the effort to avoid abandonment, and identity disorder, plays a pivotal role in the maintenance of core symptoms of BPD and represents a fundamental feature in BPD diagnosis.

## Discussion

In the present systematic review, we evaluated the role of emotional instability in BPD to stress the negative impact that the dysregulation of this cognitive process can have on daily-life functioning. Overall, the literature of the last decade confirmed an intrinsic relationship between emotional instability and BPD. Indeed, a large number of researches examined have indicated how, in individuals with BPD, this impaired cognitive function contributes to making worse the disease, both in the young and elderly. Moreover, this BPD feature represents, among other things, one of the risk factors potentially involved in the disease onset [48].

In light of this, based on the neural correlates of emotional process and BPD [29, 49, 50], future studies could aim at developing ad hoc cognitive assessment protocols for the early detection of emotional process impairment. The functional neuroimaging studies conducted to investigate emotional processing in BPD showed that emotional instability in BPD is related to a specific dysregulation activity pattern that results in a decrease in prefrontal cortex activation and enhanced insula and amygdala activity [51, 52]. Moreover, the structural MRI studies suggest that patients with BPD, with respect to control, showed a decreased volume in the amygdala, orbitofrontal cortex, anterior cingulate cortex, and hippocampus [53, 54], brain areas typically involved in emotional stimuli processing and emotional response elaborating.

Considering that emotional instability and maladaptive emotional responses define the dysregulation of affective in BPD and that this dysfunction pattern may appear in early periods of development [55–57], future studies could clarify the specific mechanism involved, during childhood, in the dysregulation of the entire emotional process. For example, Selby et al. [58] highlighted that the perception of emotional parental invalidation during childhood mediates the relationship between BPD features and romantic relationship dysfunction. However, more data are needed to explain both the development and maintenance mechanisms of the disease.

With regard to the development of novel cognitive tasks to assess the emotional functioning in BPD, an interesting finding emerged in Huben et al. [41, 42]. The authors introduce the “emotional switching” process in BPD, describing how the rapidly emotional switch from negative to positive affect and vice versa. Changing emotional state, also change mental set. The shift from different mental set is mediated by the process of executive attention and attentional switching [59–61]. Typically, one of the most widely used paradigms for the study and measurement of mental set-shifting processes is task-switching [62]. A modified version of the task-switching protocol that includes emotionally connoted stimuli [63] could be implemented for the assessment of the “emotional switching” in BPD.

## Conclusions

Taken together, evidence accumulated so far supports the pivotal role of emotional instability in the development and maintenance of BPD: the impaired processing of emotional and environmental (i.e., stressors) stimuli negatively mediate the behavior of the patients with BPD, with a strong impact on daily-life functioning.

The pervasive symptomatology pattern of this mental disorder, in which the emotion instability represents a core and stable disease feature, represent an important public health problem. A large body of literature highlights a prevalence of BPD between 0.2 and 1.8%, [64, 65] reporting the presence of suicidal behavior in 69–80% of patients [66]. These data confirm the importance of assessing the emotional impairment in BPD, in order to identify the specific features it assumes in this mental disorder and detect focused therapies can early improve the global functioning of these patients.

To this end, the development of new neuropsychological assessment tools could help clinicians in planning better therapeutic strategies and patient management.

### Strengths and limitations

The present review reported the results of the complex relationship between BPD and emotional instability, with a, update to the previous review [52]. The strength of this review is given by the methodological approach used during all steps, from data collection to the quality assessment of included studies.

The limitation of the present review may be represented by the inclusion of the studies that have assessed patients with BPD during COVID-19 pandemic. This aspect could have modulated, through complex mechanisms still under examination, the entire emotional process and, specifically, the features of emotional dysregulation in BPD.

## Supplementary Information

**Additional file 1.** Search Line Syntax.

**Additional file 2.** Table of contents.

## Data Availability

All data generated or analyzed during this study are included in this published article [and its additional information files].

## Abbreviations

DSM-V: Diagnostic and Statistical Manual of Mental Disorders, 5th edition
BPD: Borderline personality disorder
PRISMA: Preferred Reporting Items for Systematic Review and Meta-Analyses
NOS: Newcastle Ottawa Scale
DERS: Difficulties in Emotion Regulation Scale

## References

[1] Damasio H, Grabowski T, Frank R, Galaburda AM, Damasio AR (1994). The return of Phineas Gage: clues about the brain from the skull of a famous patient. Science.

[2] Koziol LF, Budding DE (2009). Subcortical structures and cognition.

[3] Ochsner K, Gross JJ, Gross J (2007). The neural architecture of emotion regulation. Handbook of Emotion Regulation.

[4] Diamond A (2013). Executive functions. Annu Rev Psychol.

[5] Zelazo PD, Müller U (2010). Executive function in typical and atypical development wiley-blackwell handb child cogn dev.

[6] Miyake A, Friedman NP, Emerson MJ, Witzki AH, Howerter A, Wager TD (2000). The unity and diversity of executive functions and their contributions to complex “frontal lobe” tasks: a latent variable analysis. Cogn Psychol.

[7] Genet JJ, Siemer M (2011). Flexible control in processing affective and non-affective material predicts individual differences in trait resilience. Cogn Emot.

[8] Bargh JA, Williams LE, Gross J (2007). On the nonconscious of emotion regulation. Handbook of emotion regulation.

[9] Diamond L, Aspinwall L (2003). emotion regulation across the life span: an integrative perspective emphasizing self-regulation, positive affect, and dyadic processes. Motiv Emot.

[10] Kotov R, Ruggero CJ, Krueger RF, Watson D, Yuan QZM (2011). New dimensions in the quantitative classification of mental illness. Arch Gen Psychiatry.

[11] Sharma A, McClellan J (2021). Emotional and behavioral dysregulation in severe mental illness. Child Adolesc Psychiatr Clin N Am.

[12] Aldao A, Nolen-Hoeksema S, Schweizer S (2010). Emotion-regulation strategies across psychopathology: a meta-analytic review. Clin Psychol Rev.

[13] Carmassi C, Bertelloni CA, Gesi C, Conversano C, Stratta P, Massimetti G (2017). New DSM-5 PTSD guilt and shame symptoms among Italian earthquake survivors: Impact on maladaptive behaviors. Psychiatry Res.

[14] Socci V, Rossi R, Talevi D, Crescini C, Tempesta D, Pacitti F (2020). Sleep, stress and trauma. J Psychopathol.

[15] Mennin DS, Holaway RM, Fresco DM, Moore MT, Heimberg RG (2007). Delineating components of emotion and its dysregulation in anxiety and mood psychopathology. Behav Ther.

[16] Brown M, Hochman A, Micali N (2020). Emotional instability as a trait risk factor for eating disorder behaviors in adolescents: Sex differences in a large-scale prospective study. Psychol Med.

[17] American Psychiatric Association. 2013 Diagnostic and statistical manual of mental disorders. Washington. DC.

[18] Putnam KM, Silk KR (2005). Emotion dysregulation and the development of borderline personality disorder. Dev Psychopathol.

[19] Zanarini MC, Frankenburg FR, Hennen J, Silk KR (2003). The longitudinal course of borderline psychopathology: 6-year prospective follow-up of the phenomenology of borderline personality disorder. Am J Psychiatry.

[20] Hua JPY, Trull TJ, Merrill AM, McCarty RM, Straub KT, Kerns JG (2020). Daily-life affective instability in emotional distress disorders is associated with function and structure of posterior parietal cortex. Psychiatry Res Neuroimaging.

[21] Lynch TR, Trost WT, Salsman N, Linehan MM (2007). Dialectical behavior therapy for borderline personality disorder. Annu Rev Clin Psychol.

[22] Lieb K, Zanarini MC, Schmahl C, Linehan MM, Bohus M (2004). Borderline personality disorder. Lancet.

[23] Stiglmayr CE, Shapiro DA, Stieglitz RD, Limberger MF, Bohus M (2001). Experience of aversive tension and dissociation in female patients with borderline personality disorder—a controlled study. J Psychiatr Res.

[24] Houben M, Claes L, Sleuwaegen E, Berens A, Vansteelandt K (2018). Emotional reactivity to appraisals in patients with a borderline personality disorder: a daily life study. Borderline Personal Disord Emot Dysregul.

[25] Koenigsberg HW, Harvey PD, Mitropoulou V, New AS, Goodman M, Silverman J (2001). Are the interpersonal and identity disturbances in the borderline personality disorder criteria linked to the traits of affective instability and impulsivity?. J Pers Disord.

[26] Migliore S, Curcio G, Porcaro C, Cottone C, Simonelli I, D’aurizio G (2019). Emotional processing in RRMS patients: dissociation between behavioural and neurophysiological response. Mult Scler Relat Disord.

[27] Maaßen E, Büttner M, Bröcker AL, Stuke F, Bayer S, Hadzibegovic J (2021). Measuring emotional awareness in patients with schizophrenia and schizoaffective disorders. Front Psychol.

[28] Cárdenas J, Blanca MJ, Carvajal F, Rubio S, Pedraza C (2021). Emotional processing in healthy ageing, mild cognitive impairment, and Alzheimer’s disease. Int J Environ Res Publ Health.

[29] Perez-Rodriguez MM, Bulbena-Cabré A, Bassir Nia A, Zipursky G, Goodman M, New AS (2018). The Neurobiology of borderline personality disorder. Psychiatr Clin North Am.

[30] Fossati A, Barratt ES, Carretta I, Leonardi B, Grazioli F, Maffei C (2004). Predicting borderline and antisocial personality disorder features in nonclinical subjects using measures of impulsivity and aggressiveness. Psychiatry Res.

[31] Krause-Utz A, Frost R, Chatzaki E, Winter D, Schmahl C, Elzinga BM (2021). Dissociation in borderline personality disorder: recent experimental, neurobiological studies, and implications for future research and treatment. Curr Psychiatry Rep.

[32] Euler S, Nolte T, Constantinou M, Griem J, Montague PR, Fonagy P (2021). Interpersonal problems in borderline personality disorder: associations with mentalizing, emotion regulation, and impulsiveness. J Pers Disord.

[33] Semerari A, Colle L, Pellecchia G, Carcione A, Conti L, Fiore D (2015). Personality disorders and mindreading. J Nerv Ment Dis.

[34] Fonagy P, Luyten P, Allison E (2015). Epistemic petrification and the restoration of epistemic trust: a new conceptualization of borderline personality disorder and its psychosocial treatment. J Pers Disord.

[35] Bateman A, Fonagy P (2010). Mentalization based treatment for borderline personality disorder. World Psychiatry.

[36] Page MJ, McKenzie JE, Bossuyt PM, Boutron I, Hoffmann TC, Mulrow CD (2020). The PRISMA statement: an updated guideline for reporting systematic reviews. BMJ.

[37] Martino F, Gammino L, Sanza M, Berardi D, Pacetti M, Sanniti A (2020). Impulsiveness and emotional dysregulation as stable features in borderline personality disorder outpatients over time. J Nerv Ment Dis.

[38] Stepp SD, Scott LN, Morse JQ, Nolf KA, Hallquist MN, Pilkonis PA (2014). Emotion dysregulation as a maintenance factor of borderline personality disorder features. Compr Psychiatry.

[39] Yen S, Frazier E, Hower H, Weinstock LM, Topor DR, Hunt J (2015). Borderline personality disorder in transition age youth with bipolar disorder. Acta Psychiatr Scand.

[40] Hepp J, Lane SP, Carpenter RW, Trull TJ (2020). Linking Daily-Life Interpersonal stressors and health problems via affective reactivity in borderline personality and depressive disorders. Psychosom Med.

[41] Houben M, Vansteelandt K, Claes L, Sienaert P, Berens A, Sleuwaegen E (2016). Emotional switching in borderline personality disorder: A daily life study. Personal Disord Theory Res Treat.

[42] Houben M, Bohus M, Santangelo PS, Ebner-Priemer U, Trull TJ, Kuppens P (2016). The specificity of emotional switching in borderline personality disorder in comparison to other clinical groups. Personal Disord Theory, Res Treat.

[43] Kobeleva X, Seidel E-M, Kohler C, Schneider F, Habel U, Derntl B (2014). Dissociation of explicit and implicit measures of the behavioral inhibition and activation system in borderline personality disorder. Psychiatry Res.

[44] Dick AM, Suvak MK (2018). Borderline personality disorder affective instability: What you know impacts how you feel. Personal Disord Theory, Res Treat.

[45] Liu Y, Zhong M, Xi C, Jin X, Zhu X, Yao S (2017). Event-related potentials altered in patients with borderline personality disorder during working memory tasks. Front Behav Neurosci.

[46] Ruocco AC, Amirthavasagam S, Choi-Kain LW, McMain SF (2013). Neural correlates of negative emotionality in borderline personality disorder: an activation-likelihood-estimation meta-analysis. Biol Psychiatry.

[47] Richetin J, Preti E, Costantini G, De Panfilis C (2017). The centrality of affective instability and identity in borderline personality disorder: evidence from network analysis. PLoS ONE.

[48] Gratz KL, Roemer L (2008). Multidimensional assessment of emotion regulation and dysregulation: development, factor structure, and initial validation of the difficulties in emotion regulation scale. J Psychopathol Behav Assess.

[49] Gan J, Yi J, Zhong M, Cao X, Jin X, Liu W (2016). Abnormal white matter structural connectivity in treatment-naïve young adults with borderline personality disorder. Acta Psychiatr Scand.

[50] Xu T, Cullen KR, Mueller B, Schreiner MW, Lim KO, Schulz SC (2016). Network analysis of functional brain connectivity in borderline personality disorder using resting-state fMRI. NeuroImage Clin.

[51] Brendel GR, Stern E, Silbersweig DA (2005). Defining the neurocircuitry of borderline personality disorder: functional neuroimaging approaches. Dev Psychopathol.

[52] Chapman AL (2019). Borderline personality disorder and emotion dysregulation. Dev Psychopathol.

[53] Wingenfeld K, Rullkoetter N, Mensebach C, Beblo T, Mertens M, Kreisel S (2009). Neural correlates of the individual emotional stroop in borderline personality disorder. Psychoneuroendocrinology.

[54] Tebartz van Elst L, Hesslinger B, Thiel T, Geiger E, Haegele K, Lemieux L (2003). Frontolimbic brain abnormalities in patients with borderline personality disorder. Biol Psychiatry.

[55] Cole PM, Llera SJ, Pemberton CK (2009). Emotional instability, poor emotional awareness, and the development of borderline personality. Dev Psychopathol.

[56] Sauer SE, Baer RA (2010). Validation of measures of biosocial precursors to borderline personality disorder: childhood emotional vulnerability and environmental invalidation. Assessment.

[57] Hill J, Stepp SD, Wan MW, Hope H, Morse JQ, Steele M (2011). Attachment, borderline personality, and romantic relationship dysfunction. J Pers Disord.

[58] Selby EA, Braithwaite SR, Joiner TE, Fincham FD (2008). Features of borderline personality disorder, perceived childhood emotional invalidation, and dysfunction within current romantic relationships. J Fam Psychol.

[59] Gilbert SJ, Shallice T (2002). Task Switching: A PDP Model. Cogn Psychol.

[60] Schneider DW, Logan GD (2009). Task switching encycl neurosci.

[61] Monsell S, Yeung N, Azuma R (2000). Reconfiguration of task-set: Is it easier to switch to the weaker task?. Psychol Res.

[62] Monsell S (2003). Task switching. Trends Cogn Sci.

[63] D’Aurizio G, Tempesta D, Saporito G, Pistoia F, Socci V, Mandolesi L (2022). Can stimulus valence modulate task-switching ability? a pilot study on primary school children. Int J Environ Res Public Health.

[64] Korzekwa MI, Dell PF, Links PS, Thabane L, Webb SP (2008). Estimating the prevalence of borderline personality disorder in psychiatric outpatients using a two-phase procedure. Compr Psychiatry.

[65] Blackburn R, Crellin MC, Morgan EM, Tulloch RMB (1990). Prevalence of personality disorders in a special hospital population. J Forensic Psychiatry.

[66] Schneider B, Schnabel A, Wetterling T, Bartusch B, Weber B, Georgi K (2008). How do personality disorders modify suicide risk?. J Pers Disord.

