# Supplementary material for: The role of emotional instability in borderline personality disorder: a systematic review
Source: Ann Gen Psychiatry. 2023 Mar 14;22:9. doi: 10.1186/s12991-023-00439-0 (PMC10011773; doi:10.1186/s12991-023-00439-0)
Supplement: Supplementary file 1 — Additional file 1. Search Line Syntax. [file 12991_2023_439_MOESM1_ESM.docx]

**S1: Search Line Syntax**

PubMed-Search: **emotional instability AND emotional dysfunction AND borderline personality disorder** Filters: **in the last 10 years, Humans, English**

(("emoting"[All Fields] OR "emotion s"[All Fields] OR "emotions"[MeSH Terms] OR "emotions"[All Fields] OR "emotion"[All Fields] OR "emotional"[All Fields] OR "emotive"[All Fields]) AND ("instabilities"[All Fields] OR "instability"[All Fields] OR "instable"[All Fields]) AND (("emoting"[All Fields] OR "emotion s"[All Fields] OR "emotions"[MeSH Terms] OR "emotions"[All Fields] OR "emotion"[All Fields] OR "emotional"[All Fields] OR "emotive"[All Fields]) AND ("dysfunctional"[All Fields] OR "dysfunctionals"[All Fields] OR "dysfunctioning"[All Fields] OR "dysfunctions"[All Fields] OR "physiopathology"[MeSH Subheading] OR "physiopathology"[All Fields] OR "dysfunction"[All Fields])) AND ("borderline personality disorder"[MeSH Terms] OR ("borderline"[All Fields] AND "personality"[All Fields] AND "disorder"[All Fields]) OR "borderline personality disorder"[All Fields])) AND ((y_10[Filter]) AND (humans[Filter]) AND (english[Filter]))

**Translations**

**emotional:** "emoting"[All Fields] OR "emotion's"[All Fields] OR "emotions"[MeSH Terms] OR "emotions"[All Fields] OR "emotion"[All Fields] OR "emotional"[All Fields] OR "emotive"[All Fields]

**instability:** "instabilities"[All Fields] OR "instability"[All Fields] OR "instable"[All Fields]

**emotional:** "emoting"[All Fields] OR "emotion's"[All Fields] OR "emotions"[MeSH Terms] OR "emotions"[All Fields] OR "emotion"[All Fields] OR "emotional"[All Fields] OR "emotive"[All Fields]

**dysfunction:** "dysfunctional"[All Fields] OR "dysfunctionals"[All Fields] OR "dysfunctioning"[All Fields] OR "dysfunctions"[All Fields] OR "physiopathology"[Subheading] OR "physiopathology"[All Fields] OR "dysfunction"[All Fields]

**borderline personality disorder:** "borderline personality disorder"[MeSH Terms] OR ("borderline"[All Fields] AND "personality"[All Fields] AND "disorder"[All Fields]) OR "borderline personality disorder"[All Fields]

Scopus-Search: TITLE-ABS-KEY ( emotional AND instability AND borderline AND personality AND disease ) AND ( LIMIT-TO ( LANGUAGE , "english" ) ) AND ( LIMIT-TO( PUBYEAR , 2022 ) OR LIMIT-TO ( PUBYEAR , 2021 ) OR LIMIT-TO ( PUBYEAR , 2020 ) OR LIMIT-TO ( PUBYEAR , 2019 ) OR LIMIT-TO ( PUBYEAR , 2018 )OR LIMIT-TO ( PUBYEAR , 2017 ) OR LIMIT-TO ( PUBYEAR , 2016 ) OR LIMIT-TO ( PUBYEAR , 2015 ) OR LIMIT-TO ( PUBYEAR , 2014 ) OR LIMIT-TO (PUBYEAR , 2013 ) OR LIMIT-TO ( PUBYEAR , 2012 ) )

Web of Science-Search: **emotional instability AND emotional dysfunction AND borderline personality disorder** (All Fields) **and Article** (Document Types) **and 2012-2022** (Publication Years) **and English** (Languages)
