# Supplementary material for: The role of emotional instability in borderline personality disorder: a systematic review
Source: Ann Gen Psychiatry. 2023 Mar 14;22:9. doi: 10.1186/s12991-023-00439-0 (PMC10011773; doi:10.1186/s12991-023-00439-0)
Supplement: Supplementary file 2 — Additional file 2. Table of contents. [file 12991_2023_439_MOESM2_ESM.docx]

| **First Author and publication year** | **Journal** | **Main objective** | **Number and characteristics of sample** | **Scales and measures** | **Main findings** |
| --- | --- | --- | --- | --- | --- |
| Martino et al., 2020 | J. Nerv. Ment. Dis. | Providing a direct comparison between younger and older BPD patients with respect to the following nine DSM-4 BPD criteria: self-harm, emotional dysregulation, impulsiveness, work and social functioning. | Young (Group 1; age 18–25 years; N = 44) and older patient (Group 2; age 40–59 years; N = 49) with BPD. | SCID-II  SHI  BIS-11  DERS  GAF | Group 1 were more likely to show anger and self-damaging behaviors compared with Group 2, which in turn were more likely to endorse chronic emptiness.  No significant differences were found in impulsiveness, social functioning, and emotional instability.  Moreover, the similar DERS scores obtained from older and younger groups showed severe impairment in the emotion regulation strategies that confirm how, independently the age of the patient, in BPD, exists a pervasive and time-independent emotional instability. |
| Richetin et al., 2017 | PLoS One | Exploring the relationship of nine BPD trait of the DSM using a network analysis, compared clinical sample with healthy controls. | Healthy controls (Group 1; age 22.56 years; N = 1317) and patient (Group 2; age 37.75; N= 96) with BPD. | BPDCL | Emotional dysfunction, effort to avoid abandonment and identity disorder play a pivotal role in the maintenance of core symptoms of BPD and represents a fundamental feature in BPD diagnosis. |
| Houben et al., 2016 | Personal Disord | Investigating the presence and specific characteristics of emotional switching between negative and positive emotional states in BPD compared with healthy control group. | 30 BPD patients (age= 29.03) and 28 healthy controls (age= 29.29). | ADP- IV  ESM | The authors found a specific effect that explains how affective instability in BPD patients is characterized by a larger "emotional switching" from negative to positive emotional states and vice versa when compared with controls. |
| Dick et al., 2018 | Personal Disord | Exploring the relationship between emotional instability, vocabulary and both valence and arousal semantic in individual with BPD and control group. | Female Patient with BPD (Group 1; N=46; age= 21.64) and female healthy control group (N=51; age= 21.64) | PANAS  AIM  SIL-V Semantic Valence and Arousal Focus | Language ability positively modulates the emotion generation process, highlighting the pivotal role of language in emotional instability and the protective role that an increased vocabulary competence could play in mitigating the negative effect of emotional dysregulation in BPD. |
| Kobeleva et al., 2014 | Psychiatry Res | Investigating the relationship between implicit and explicit measure of socioemotional processing in BPD. To measure implicit behaviour after negative emotional facial expression in BPD patients with respect to control group. | Patients with BPD (N=25; age = 26.8) and healthy controls (N=25; age = 26.88) | Rating task  Joystick task  VERT-K  MWT-B  TMT A/B  RWT  Digit Span  PANAS  RSQ  BDI | The authors found a significant difference between BPD and control groups showing emotion-specific alteration during the evaluation of happy and fearful faces in the BPD patients. |
| Hepp et al., 2014 | Psychosom Med | Exploring the temporal relationship between negative affect and physical health problem and potential time-gradient of this association in BPD. | BPD (age= 18-65 years; N=81) and depressed participants (DD; age= 18-65 years; N=50) | PANAS-X, 24  Physical Symptom Checklist | Altered and non-adaptive emotional responses to interpersonal and environmental stressors, associated with emotional instability, may explain unstable interpersonal relationships observed in BPD. This feature of BPD becomes even more evident when subjects have to process negative stimuli. These impairments in turn may contribute to an increase in the severity of BPD symptoms. |
| Stepp et al., 2014 | Compr. Psychiatry | Investigating emotional dysregulation as a maintenance factor for BPD characteristics over the course of one years. | Patients with BPD (age= 45; N=75) and control group (age= 21-60; N=75) | SIDP-IV  PAI-BOR  DERS  CTS2  ARS | The results showed an increase in emotional dysregulation. This factor seemed to mediate the BPD severity symptoms at baseline and at 12-month follow-up. Furthermore, emotional instability appears to be a maintenance mechanism of the BPD. |
| Yen et al., 2015 | Acta Psychiatr. Scand. | Exploring the longitudinal impact of features and severity of BPD on the course of the disease and if these characteristics predict a more pernicious course of bipolar disorder into adulthood. | Patients with BPD (N= 271; age= 20.65) | SIDP-IV  K-SADS-PL  KSADS-MRS  K-DEP  LIFE  PSR  CGAS  SCARED  CBCL  YSR  FACES-II  CBQ | BPD symptoms negatively predicts the clinical disease course. Interestingly, a stronger association emerges when considering affective dysregulation. These results confirm the centrality of emotional dysregulation in BPD. |
| Houben et al., 2016 | Personal Disord | Investigating emotional switching in patients with PBD, depressive disorder, bulimia nervosa, and post-traumatic stress disorder. | 2 samples by comparing BPD patients (N = 43 in sample 1; N = 81 in sample 2) to patients with bulimia nervosa (N = 20), post-traumatic stress disorder (N = 28), or healthy controls (N = 28) in sample 1, and to patients with depressive disorder (N = 50) in sample 2. | PANAS  ESM | Emotional switching might reflect a feature of emotional response, characterizing a range of disorders with mood disturbances. |
| Ruocco et al., 2013 | Biol Psychiatry | Using Likelihood-Estimation Meta-Analysis, synthesizing neuroimaging studies of negative emotionality and emotional instability in BPD to detect the neural structures that show functional abnormalities in this mental disorder. | Patients with BPD (N=145) and healthy control subject (N=150). | n.a. | During processing of negative emotions, BPD patients showed, with respect to healthy control group, greater activation in the insula and posterior cingulate cortex and less activation of a neural network extending from subgenual anterior cingulate and dorsolateral prefrontal cortex to the amygdala. |
| Liu et al., 2017 | Front. Behav. Neurosci | Using ERP-s, explore emotional instability, impulsiveness, and working memory in BPD group with respect to healthy controls. | Patients with BPD (age= 22-25; N= 22) and age and sex-matched healthy control (N= 21). | CES-D  BIS-11  CTQ  SAIS  N-back task | BPD patients showed lower P3 amplitudes and longer N2 latencies than healthy control suggesting a dysfunction on working memory sub-processes, but not during negative emotional processing. |

S2: Included studies and main extracted data. Note. SCID-II= Structured Clinical Interview for DSM-4 Axis II Personality Disorder; SHI= Self-Harm Inventory; BIS-11= The Barratt Impulsiveness Scale; DERS= Difficulties in Emotion Regulation Scale; BPDCL= Borderline Personality Disorder Checklist; GAF= Global Assessment of Functioning; PANAS= Positive and Negative Affect Schedule; AIM= Affect Intensity Measure; SIL-V= The Shipley Institute of Living Scale, Vocabulary Subscale; ESM= Experience-Sampling Method; VERT-K= The emotion recognition task; MWT-B= Mehrfachwahl-Wortschatz-Intelligent-Test; SIL-V= The Shipley Institute of Living Scale, Vocabulary Subscale; TMT= Trail Making Test Part A/B; RWT= Regensburg Word Fluency Test; RSQ= Relationship Scales Questionnaire; BDI= Borderline Personality Inventory; SIDP-IV= Structured Interview for DSM-IV Personality; PAI-BOR= Personality Assessment Inventory – Borderline Features Scale; ARS= Anger Rumination Scale; ADP- IV= The Assessment of DSM–IV Personality Disorders Borderline personality disorder scale; PAI-BOR= Personality Assessment Inventory – Borderline Features Scale; CTS2= Revised Conflict Tactics Scale; ARS= Anger Rumination Scale; K-SADS-PL= Schedule for Affective Disorders and Schizophrenia for School-Age Children—Present and Lifetime Version; KSADS-MRS = Kiddie Mania Rating Scale; K-DEP= Depression Rating Scale; LIFE= Longitudinal Interval Follow-up Evaluation; PRS= Psychiatric Status Rating; CGAS= Children's Global Assessment Scale; SCARED= Child Anxiety Related Emotional Disorders; CBCL = Child Behavior Checklist; YSR= Youth Self Report; FACES-II= Family Adaptability and Cohesion Evaluation Scales-II; CBQ = Conflict Behavior Questionnaire; CES-D= Center for Epidemiological Studies Depression Scale; CTQ= Childhood Trauma Questionnaire; SAIS= Short Affect Intensity Scale; PANAS-X, 24= Positive and Negative Affect Schedule-Extended version; DSM= Diagnostic and statistical manual of mental disorders; ERP-s= event-related potentials; n.a.= not applicable.
